# Pyridostigmine Restores Cardiac Autonomic Balance after Small Myocardial Infarction in Mice

**DOI:** 10.1371/journal.pone.0104476

**Published:** 2014-08-18

**Authors:** Marina T. Durand, Christiane Becari, Mauro de Oliveira, Jussara M. do Carmo, Carlos Alberto Aguiar Silva, Cibele M. Prado, Rubens Fazan, Helio C. Salgado

**Affiliations:** 1 Department of Physiology, School of Medicine of Ribeirão Preto, University of São Paulo, Ribeirão Preto, São Paulo, Brazil; 2 Department of Pathology, School of Medicine of Ribeirão Preto, University of São Paulo, Ribeirão Preto, São Paulo, Brazil; 3 Department of Physiology and Biophysics, University of Mississippi Medical Center, Jackson, Mississippi, United States of America; University of Buenos Aires, Faculty of Medicine, Cardiovascular Pathophysiology Institute, Argentina

## Abstract

The effect of pyridostigmine (PYR) - an acetylcholinesterase inhibitor - on hemodynamics and cardiac autonomic control, was never studied in conscious myocardial infarcted mice. Telemetry transmitters were implanted into the carotid artery under isoflurane anesthesia. Seven to ten days after recovery from the surgery, basal arterial pressure and heart rate were recorded, while parasympathetic and sympathetic tone (ΔHR) was evaluated by means of methyl atropine and propranolol. After the basal hemodynamic recording the mice were subjected to left coronary artery ligation for producing myocardial infarction (MI), or sham operation, and implantation of minipumps filled with PYR or saline. Separate groups of anesthetized (isoflurane) mice previously (4 weeks) subjected to MI, or sham coronary artery ligation, were submitted to cardiac function examination. The mice exhibited an infarct length of approximately 12%, no change in arterial pressure and increased heart rate only in the 1st week after MI. Vagal tone decreased in the 1^st^ week, while the sympathetic tone was increased in the 1^st^ and 4^th^ week after MI. PYR prevented the increase in heart rate but did not affect the arterial pressure. Moreover, PYR prevented the increase in sympathetic tone throughout the 4 weeks. Concerning the parasympathetic tone, PYR not only impaired its attenuation in the 1^st^ week, but enhanced it in the 4^th^ week. MI decreased ejection fraction and increased diastolic and systolic volume. Therefore, the pharmacological increase of peripheral acetylcholine availability by means of PYR prevented tachycardia, increased parasympathetic and decreased sympathetic tone after MI in mice.

## Introduction

Myocardial infarction (MI) as a result of coronary artery occlusion is always accompanied by marked changes in cardiac autonomic control characterized by overall sympathetic activation and cardiac parasympathetic attenuation, contributing to progressive ventricular dysfunction [1], [2]. While species such as dog, rabbit and rat have been studied exhaustively as models of autonomic dysfunction in heart failure, only recently the mouse has attracted much attention. This is because the mouse has been routinely genetically manipulated and is a reference in studies of the molecular factors involved in physiological functions and several diseases involving the cardiovascular system [3], for instance the MI. Moreover, the development of telemetric recording for mice allowed the evaluation of long term oscillations of arterial pressure (AP) and heart rate (HR), providing a reliable and powerful tool to exam the mechanisms of cardiovascular control in freely moving subjects for long periods [4], [5].

Derangement of vagal control of the HR becomes apparent at early stage of left ventricular dysfunction [6], [7] and is closely associated with poor long-term outcome and high mortality of patients following MI, or development of heart failure [8], [9]. In addition, recent studies demonstrated that chronic disturbance of the cholinergic tone in genetically manipulated mice, i.e. acetylcholine receptor knockout or vesicular acetylcholine transporter knockdown, elicited a decrease in left ventricular performance associated with altered calcium handling and plastic alterations contributing to heart dysfunction [10], [11]. Therefore, activation of parasympathetic function may have beneficial implications after acute cardiac ischemic injury or further development of heart failure [12], [13]. Previous studies have demonstrated that chronic vagal stimulation improved ventricular performance, reduced cardiac remodeling and arrhythmias, and improved the cardiac autonomic control, lengthening long-term survival in experimental models of heart failure [14], [15]. In patients with heart failure, vagus nerve stimulation carried out during a six-month period was associated with significant improvement in NYHA class, quality of life, 6-minute walk test, left ventricular ejection fraction and systolic volume [13]. Therefore, the increase in parasympathetic function seems to be a promising therapeutic alternative for preventing MI outcomes, by acting on the autonomic control of the heart and improving cardiac remodeling and function.

However, vagal nerve stimulation therapy is an invasive approach, whose feasibility is under active investigation [16], [17]. Because the efferent vagal nerve activity is ultimately mediated by acetylcholine, modulation of this neurotransmitter, by means of pharmacological agents that augment the acetylcholine availability at the neuroeffector junction, should be considered a promising therapy for patients with MI and/or heart failure. Anticholinesterase agents have been used in experimental designs aiming to enhance the parasympathetic influences on the heart [18]. Pyridostigmine (PYR), a reversible anticholinesterase agent that does not cross the blood-brain barrier and acts particularly in the peripheral synaptic cleft, seems to be potentially useful in this context.

Previous studies have demonstrated that short-term administration of PYR may be potentially useful for cardiovascular protection because it shifts the autonomic balance toward parasympathetic predominance, resulting in beneficial effects on markers of cardiovascular risk and dysfunction [19]–[22]. PYR increased HR variability in normal rats [23], healthy humans [24] and patients with heart failure [25]. In humans, the acute administration of PYR produced bradycardia [26], increased the ventricular refractory period and displayed a potential anti-arrhythmic effect at higher HR [27]. In patients with coronary artery disease and heart failure, PYR prevented the myocardial dysfunction induced by mental stress [19], ameliorated the autonomic and hemodynamic profile during exercise [28]–[30], improved the HR recovery after exercise [31] and reduced ventricular arrhythmia density [25]. Moreover, our laboratory has observed a protective effect of PYR on cardiac autonomic control, intrinsic HR, baroreflex sensitivity, and cardiac function four [20] and six weeks [21] after the onset of heart failure in rats.

To our knowledge, the chronic effect of PYR administration has never hitherto been studied in mice with MI, a species amenable to genetic manipulation which has becoming a reference in studies of physiological functions and pathophysiology of many diseases, for instance MI. Therefore, the objective of the current study was to evaluate, by means of telemetry in conscious mice, whether the increase of parasympathetic function by with PYR administration improves the hemodynamics (AP and HR) and sympathovagal balance, one and four weeks after MI elicited by coronary artery ligation.

## Methods

### Animals

Experiments were carried out in male C57/BL6 mice, 10–15 weeks of age, averaging 25–30 g, supplied by the Animal Facility of the School of Medicine of Ribeirão Preto, University of São Paulo (Ribeirão Preto, SP, Brazil). The mice were fed a standard chow diet and tap water *ad libitum*, and were housed under controlled temperature (22°C) with a 12 h dark-light cycle. All procedures were approved by the Committee of Ethics in Animal Research of the Medical School of Ribeirão Preto, University of São Paulo (Ribeirão Preto, SP, Brazil) [Protocol # 203/2008].

### Telemetry probe implantation

Mice were anesthetized with isoflurane (5% for induction and 2–2.5% for maintenance) diluted in 100% oxygen. The anesthetic mixture was provided via an isoflurane vaporizer (*Calibrated Vaporizer Takaoka, model ISOVAPOR 1224, K. Takaoka Ind. Com. Ltda., São Paulo-SP, Brazil*), which delivered the anesthetic mixture into a mask. Prior to implantation, the telemetry probes were carefully prepared with application of a biocompatible gel on the tip of the catheter, to prevent blood from entering the catheter lumen; following, the transmitter calibration was proceeded. Surgical procedures were performed under aseptic conditions and under magnification by a surgical microscope (*CM-A199 DFZ, DF Vasconcellos SA Optics and High Precision Mechanics, Valencia, RJ*). The animals were implanted with radio telemeter probes (*TA11PA-C10, Data Sciences International, St. Paul, MN*), as described by Butz and Davisson [5]. Briefly, after shaving the ventral neck region with a depilatory cream and disinfecting the skin, a midline incision was made and the left carotid artery was isolated by blunt dissection. After cranial permanent ligature and momentary caudal occlusion with a thread, the catheter of the radio telemeter was inserted into the carotid and the same thread was used to secure the catheter. Through the ventral neck incision, a subcutaneous pouch was formed for placement of the transmitter body along the animal's right flank. The skin was then closed with a 6-0 silk suture.

### Myocardial infarction

MI was performed as described elsewhere [2]. Briefly, under the same anesthetic protocol described above, a ventral midline skin incision was made caudally to the larynx to access the trachea; then, a small incision was made in the trachea to introduce a 22G needle tip (*BD Angiocath Becton Dickinson Ind. Cir. Ltda., Juiz de Fora, MG, Brazil*) which was connected to a rodent ventilator (*Inspira Advanced Safety Single Animal Pressure/Volume Controlled Ventilator, Harvard Apparatus, Holliston, MA, USA*). The rodent ventilator acquired the anesthetic mixture from the isoflurane vaporizer. An 80-µL tidal volume was maintained at 70 cycles per minute. After the skin was shaved and disinfected, the 3^rd^ intercostal space was opened, the heart was visualized and the pericardium was gently opened. The left anterior descending coronary artery was visualized and ligated with 8-0 polypropylene suture. The intercostal incision was closed with a 6-0 silk suture, and a 21G needle attached to a 3.0-mL syringe was introduced through the closed wound and the negative pressure of the thorax was reestablished avoiding damage to heart and lungs. Finally, the skin was closed with 6-0 silk suture. To prevent subsequent infection, 50 µL of a mixture of penicillin and streptomycin (*Pentabiótico Veterinário Pequeno Porte, Fort Dodge Saúde Animal, Campinas, SP, Brazil*) was administered intramuscularly. The infarct size was confirmed by postmortem examination. Control animals were submitted to sham surgery, in which all the steps, except the left anterior descending coronary artery ligation, were performed. All surgical procedures were carried out under microscope magnification (*CM-A199 DFZ, DF Vasconcellos SA Optics and High Precision Mechanics, Valencia, RJ*).

### Implantation of mini pump for subcutaneous administration of pyridostigmine

Under the same anesthesia used to perform the MI an osmotic minipump (model 1004, volume of 0.11 µL/h over 28 days; *DURECT - ALZET Osmotic Pumps, Cupertino, CA, USA*) was implanted subcutaneously just below the scapular region. Following, the animal was released from the anesthesia and placed in an individual cage for recovery. The minipumps were prefilled with PYR (*Valeant Pharmaceuticals in Brazil Ltda, Campinas, SP, Brazil*), or vehicle (sterile NaCl 0.9%), and primed by incubation under isotonic saline solution at 37°C for 48 h, according to manufacturer's instructions. PYR was subcutaneously supplied at 3 mg/kg/day for four weeks.

### Experimental Protocol 1

After the probe implantation, the animals were allowed to recover for 7 to 10 days before basal recordings. Basal AP and HR signals were sampled (2 kHz) continuously for two hours. After basal recording, the mice received methyl atropine (2 mg/kg, i.p.) and propranolol (5 mg/kg, i.p.) separated by a 40 to 50 min interval. The AP and HR recording was maintained for at least 30 minutes after the blockade of the autonomic receptors. After 24 hours, the protocol was repeated with the autonomic blockers administered in reverse order, i.e. propranolol followed by methyl atropine. On the next day, a surgical procedure to produce MI and minipump implantation was carried out. The animals were randomly sorted to receive minipump filed with PYR (n = 7) or isotonic saline solution (n = 8). The protocol for data acquisition, as described above, was repeated one and four weeks after MI. The data from the telemetry device were collected by means of a receiver placed under the mouse cage (Dataquest, *Data Sciences International, Saint Paul, MN*).

### Experimental Protocol 2

This protocol was carried out in separate groups of anesthetized mice previously (4 weeks) subjected to MI, or sham left anterior descending coronary artery ligation, and implanted with osmotic minipumps filed with PYR or saline, as described above. The mice were anesthetized by inhalation of isoflurane (5% for induction and 2–2.5% for maintenance). A microtip pressure-volume catheter (*SPR-839, Millar Instruments, Houston, TX, USA*) was inserted into the right carotid artery for measurement of the AP and then moved into the left ventricle as described elsewhere [32]. After stabilization, the signals were continuously recorded using the pressure-volume conductance system (*MPVS, Millar Instruments, Houston, TX, USA*) connected to a Power Lab/4SP (*ADI Instruments, Sydney, NSW, Australia*) feeding the digital signal to an IBM PC. The HR, left ventricular end-systolic pressure (LVESP), left ventricular end-diastolic pressure (LVEDP), maximal slope of the systolic pressure increment (+dP/dt) and diastolic pressure decrement (−dP/dt), end-systolic volume (ESV), end-diastolic volume (EDV), stroke volume (SV), ejection fraction (EF) and cardiac output (CO) were calculated using a cardiac pressure-volume analysis program (*PVAN Ultra 1.1, Millar Instruments, Houston, TX, USA*) as described elsewhere [32]. The CO was normalized to body weight [cardiac index (CI)]. The total peripheral resistance index (TPRI) was calculated by the following equation: TPRI = MAP/CI.

### Morphological analysis

After the hemodynamic recordings, the animals were killed with an overdose of ketamine and xylazine (1∶1; *Ketamina Agener União Saúde Animal, Embu-Guaçu, SP, Brazil; Dopaser Hertape Calier Saúde Animal S/A, Juatuba, MG, Brazil*) and the whole hearts were harvested, rinsed in 10% KCl solution for stopping the heart in diastole, and weighed. The hearts were cut transversely and fixed in phosphate-buffered 10% formalin for 24 to 48 hours. For histological processing the hearts were embedded into paraffin and each block was serially cut with 6 µm thickness. The sections were stained with picrosirius red or hematoxylin and eosin. For infarct size and volume fraction (%) of collagen analysis the hearts stained with picrosirius red were used. Assessment of the infarct size was performed according to Takagawa et al. [33]. Briefly, the infarct size (%) was calculated by dividing the infarct length - between the surfaces of the epicardium and endocardium - by the circumference of the left ventricle and multiplying by 100. To estimate the volume fraction (%) of collagen in picrosirius red-stained sections, quantitative examination of the surviving left ventricular was carried out on a medium-power light-microscopic field. For each heart, 15 fields per mice were randomly selected and the mean value was subsequently calculated. For morphometric analysis the hearts stained with hematoxylin and eosin were used and the minor diameter of myocytes in the surviving left ventricle was measured. Since the myocardial fiber has a tubular shape, the smallest axis is a measure perpendicular to the nucleus, no matter the myocardial fiber is sectioned in longitudinal, oblique or transversal manner. This is the right diameter of the structure [34]. Approximately 30 values were obtained per mice, and the mean value was calculated. The sections were analyzed using the video microscopy software Leica Qwin (*Leica Imaging Systems, Cambridge, UK*) and the public-domain software NIH ImageJ (*developed by U.S. National Institutes of Health and available at* http://rsb.info.nih.gov/nih-image/).

### Data analysis

Pulsatile AP recordings were analyzed by customized computer software designed to detect inflection points of a periodic wave. Beat-by-beat time series of systolic AP were generated. Series of pulse intervals were obtained by measuring the time-interval between two consecutive systolic AP values.

**Cardiac autonomic tone and intrinsic HR:** Tonic sympathetic and vagal influences on the heart were assessed by HR changes induced by autonomic blockade produced by propranolol and methyl atropine, respectively. The difference between the HR measured at the end of the 40-min time-frame after propranolol administration and basal HR was considered the sympathetic tone. The difference between the HR measured at the end of the 40-min time-frame after methyl atropine administration and basal HR was considered the vagal tone. HR values obtained after the double autonomic receptor blockade provided the intrinsic HR, i.e. the HR remaining after the removal of adrenergic and cholinergic influence upon the heart.

### Statistical Analysis

The data are expressed as the mean ± standard error of the mean (SEM). Comparisons of the general characteristics of the animals studied, morphological and cardiac function parameters were performed using two-way analysis of variance (ANOVA). For multiple comparisons of hemodynamic data over time (before, one week and four weeks), two-way ANOVA for repeated-measures analysis were performed. Data from cardiac sympathetic and vagal tone were compared among groups using one-way ANOVA followed by the Student-Newman-Keuls *post-hoc* comparisons. The level of significance was set as p<0.05.

## Results

### General characteristics of control and myocardial infarcted mice with or without pyridostigmine treatment

**Figure 1:**
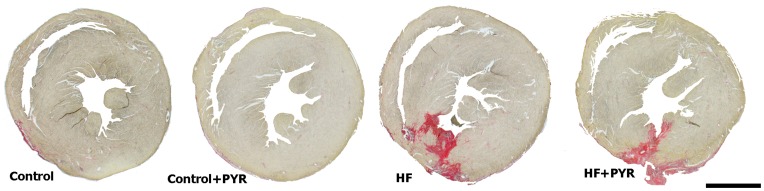
Cross sections of representative hearts, stained with picrosirius red, from control and mice with myocardial infarction (MI), treated, or not, with pyridostigmine (PYR). Bar = 1 mm.

Representative heart sections of control and myocardial infarcted mice treated, or not, with PYR are shown in Figure 1. Remarkable scarring can be observed in the left ventricle of myocardial infarcted mice, while the control animals display normal left ventricular wall without damage to the myocardium.

**Figure 2:**
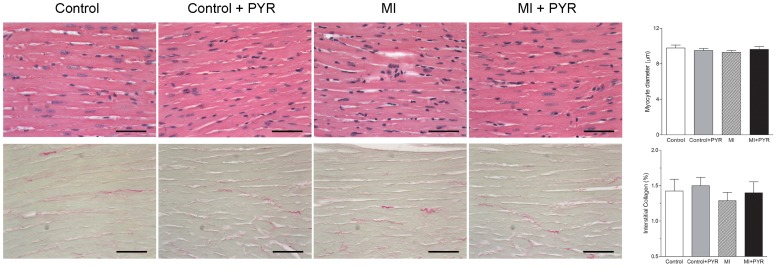
Photomicrographs and bar graphs of the minor diameter of myocytes and collagen density from the left ventricle of control and myocardial infarcted mice (MI) with or without pyridostigmine (PYR) treatment. Values are presented as the mean ± SEM. Bar = 60 µm.

The general characteristics of control and myocardial infarcted mice, with or without PYR treatment, are shown in Table 1. Body weight was similar in the four groups. However, heart weight and cardiac index were increased only in myocardial infarcted mice treated with PYR. The infarct size was similar between myocardial infarcted mice with and without PYR treatment. Nevertheless, Figure 2 shows that myocytes size and collagen were similar between groups.

**Table 1:** General characteristics of control and myocardial infarcted (MI) mice with or without pyridostigmine (PYR) treatment.

	Control	Control+PYR	MI	MI+PYR
	(n = 6)	(n = 7)	(n = 13)	(n = 15)
**Body weight (g)**	29±1	30±1	30±0.5	31±1
**Heart weight (mg)**	119±3	118±4	124±3	135±3* #
**Cardiac Weight Index (mg/g)**	4.1±0.1	3.9±0.1	4.2±0.1	4.4±0.1*
**Infarct size (% of left ventricle)**	–	–	13±1	11±1

Values are presented as mean ± SEM;

*p<0.05 compared to control+PYR group.

# p<0.05 compared to MI group.

**Figure 3:**
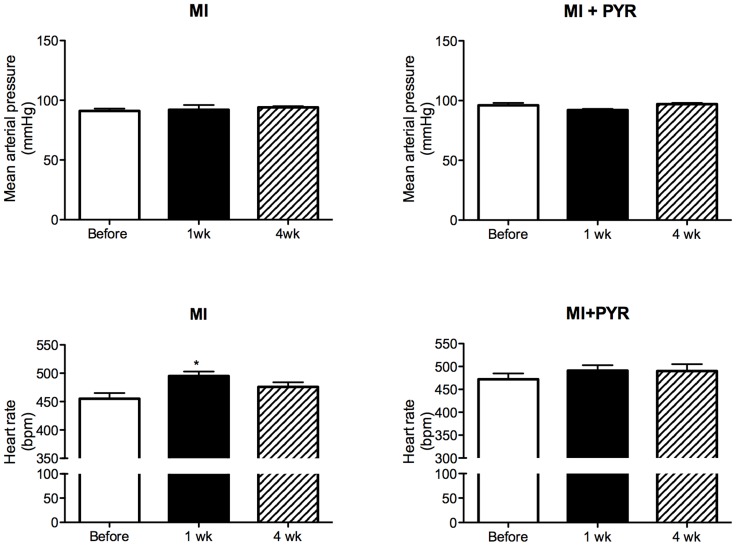
Mean arterial pressure and heart rate obtained before, one week (1 wk) and four weeks (4 wk) after myocardial infarction (MI) in mice treated with saline [n = 8] or pyridostigmine (PYR) [n = 7]. Values are presented as mean ± SEM. *p<0.05 compared to before.

Figure 3 shows that MI combined, or not, with PYR did not change the mean AP. Nevertheless, one week after MI it was observed an increase in HR which was back to normal levels - before - in the fourth week. Moreover, the increase in HR observed one week after the MI was prevented by PYR.

### Cardiac autonomic tone and intrinsic heart rate

**Figure 4:**
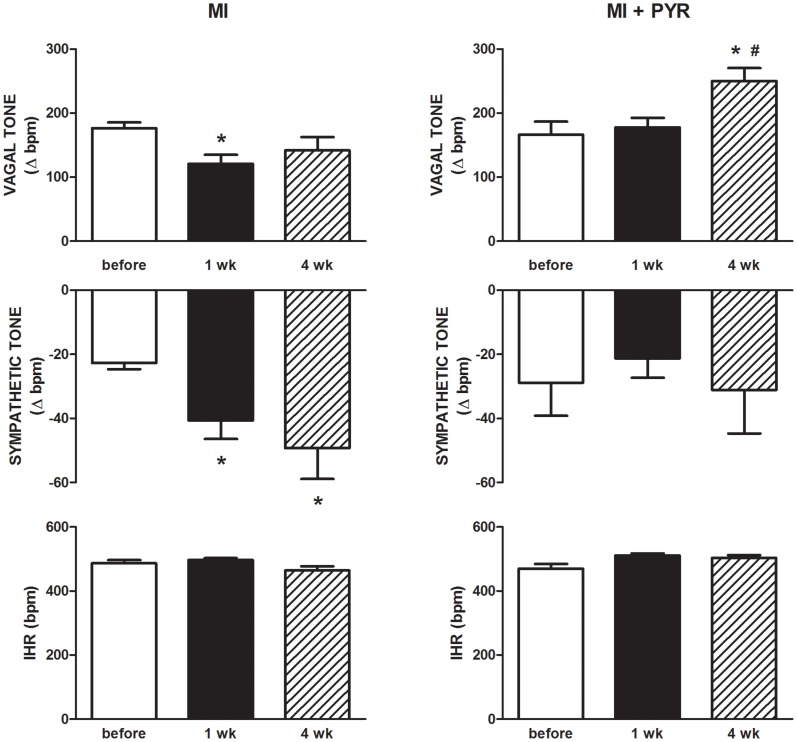
Tachycardic response caused by methyl atropine (Vagal Tone) [left and right upper panel], bradycardic response caused by propranolol (Sympathetic Tone) [left and right middle panel] and intrinsic heart rate (IHR) [left and right lower panel] before, one week (1 wk) and four weeks (4 wk) after MI with or without pyridostigmine (PYR) treatment. Values are presented as mean ± SEM. *p<0.05 compared to before; #p<0.05 compared to one week. MI: n = 8; MI+PYR: n = 7.

Figure 4 (left upper panel) shows that one week after MI it is observed a significant decrease in vagal tone influence to the heart when compared with the observations before the ligation. However, this reduction in vagal tone was not maintained at four weeks after MI. In contrast, conscious mice showed significant increase in sympathetic tone in both one and four weeks after MI (Figure 4, left middle panel). PYR (Figure 4, right upper panel) not only prevented the decrease in vagal tone one week after MI but also increased this parameter in the fourth week as compared to observations from before MI. In addition, PYR also prevented the increase in sympathetic tone at one and four weeks after MI (Figure 4, right middle panel). The combined administration of methyl atropine and propranolol shows that neither MI (Figure 4, left lower panel) nor PYR (Figure 4, right lower panel) affected the intrinsic HR of the mice.

### Hemodynamics and left ventricular function

In the groups used for cardiac function studies under isoflurane anesthesia, the infarct size in mice treated with PYR (11±1%) was the same as in those without PYR treatment (13±1%). Table 2 shows the hemodynamic parameters and the indices of systolic and diastolic function from P-V relations. The hemodynamic parameters, i.e. mean AP and HR were similar in the four groups studied. The parameters obtained from P-V analysis showed that mice with MI presented after four weeks a mild decrease in cardiac function, expressed by the increase in left ventricular end-systolic and end-diastolic volumes and lower ejection fraction. All other parameters expressing cardiac function were unaltered four weeks after MI. PYR did not affect the small alterations of cardiac function in myocardial infarcted mice.

**Table 2:** Hemodynamic parameters and indices of left ventricle function derived from pressure-volume recordings in isoflurane anesthetized control and myocardial infarcted (MI) mice with or without pyridostigmine (PYR) treatment.

	Control	Control+PYR	MI	MI+PYR
	(n = 6)	(n = 7)	(n = 5)	(n = 8)
**MAP (mmHg)**	81±3	79±3	74±4	85±3
**HR (bpm)**	492±14	457±33	503±29	527±16
**+dP/dt (mmHg/s)**	9393±631	8958±574	9285±487	8133±619
**−dP/dt (mmHg/s)**	−8845±864	−8677±1222	−9133±282	−6742±645#
**LVEDP (mmHg)**	3.5±1.3	5.7±2.0	5.0±1.3	7.6±2.6
**ESV (µL)**	11.6±0.9	15.1±3.7	24.1±2.5*	20.2±2.1
**EDV (µL)**	17.8±1.7	24.1±5.0	31.4±2.4*	29.5±3.7
**SV (µL)**	7.3±1.1	9.0±1.6	7.3±1.5	9.4±2.1
**EF (%)**	39±3	39±4	24±5*	29±5
**CO (µL/min)**	3583±579	3904±686	3785±904	4859±1084
**CI (mL/min/kg)**	106±18	117±20	115±27	147±34

Values are presented as the mean ± SEM.

*p<0.05 compared control group.

# p<0.05 compared to MI group.

## Discussion

It was demonstrated that the increased availability of peripheral acetylcholine after blocking the acetylcholinesterase with PYR, prevented tachycardia, increased parasympathetic and decreased sympathetic tone after MI in mice.

The small infarct length (∼12%) did not cause changes in AP but led to a rise in basal HR in the first week after MI. The literature does not have a consensus concerning the responses of AP and HR after MI. Overall, it has been shown that an infarct size of 20 to 30% in mice does not affect the HR [35]–[37], although the AP may be decreased [35], [37] or unchanged [36]. Thus, it is possible that different methodological approaches for recording the AP (intra-arterial cannula, indirect measurement from tail cuff and telemetry), as well as different types of anesthesia and time frame after MI have significant influences on the AP and HR recorded. Nevertheless, hemodynamic long-term telemetric recording in conscious mice demonstrated that even a relatively small MI caused tachycardia in the first week after the coronary artery ligation, which was prevented by PYR.

The extent of observed MI was enough to induce mild impairment in cardiac performance and significant changes in autonomic control of the heart. Apropos, in humans the median infarct size of silent MI involves 10% to 12% of the left ventricle, with a median left ventricular ejection fraction of 47% [38]. Of note, as observed in humans, the left ventricular function declines with the length of the infarcted area in mice [35]. Acute MI in mice has been thoroughly examined [1], [2], [33], but the characterization of the relationship between survival and extent of the infarcted area is considered a difficult task. Sallto-Tellez et al. [2] demonstrated that the histological changes found in myocardial infarcted mice are similar to those observed in humans, highlighting the importance of this model for translational studies.

Clinical and experimental studies have shown that MI followed by heart failure is combined with reduced cardiac vagal tone, which appears early in the development of this outcome, preceding sympathetic activation [8], [39], [40]. In patients and experimental models of heart failure, blockade of muscarinic receptor has a modest effect on HR compared to healthy subjects [20], [21], [39]–[41]. In the present study, the assessment of the cardiac autonomic balance showed reduced vagal tonic influence to the heart in the first week post MI, and increased cardiac sympathetic drive in the first and fourth week post MI. Thus, this autonomic imbalance may certainly be responsible for the increased HR observed one week after MI, because no alteration was observed in the intrinsic HR itself. At four weeks after MI, reduction of the vagal tone was not as evident as in the first week, which may explain the return of HR to baseline levels.

PYR was able to reduce the loss of vagal and the increase of the sympathetic cardiac tone at four weeks after MI. PYR has produced likewise a great increase in vagal tone at four weeks post MI, supporting the notion that the blockade of acetylcholinesterase was effective in preserving the cardiac autonomic balance in myocardial infarcted subjects. Previous studies have provided similar results, demonstrating that short- and long-term treatment with PYR reduced basal HR and prevented the reduction in the vagal tone and the increase of the sympathetic tone in the early and late phases of heart failure following MI in rats [20]–[22]. The increase in parasympathetic activity produced by PYR may be attributed to the potentiation of cholinergic neurotransmission. It has been shown that the same dose of PYR used in the present study (3 mg/kg) inhibited 85% of acetylcholinesterase activity one week after its administration, increasing the permanence of acetylcholine in the synaptic cleft [42].

The neural control of the heart is exerted by the tonic interaction between the sympathetic and parasympathetic arms of the autonomic nervous system [43]. Studies have demonstrated that acetylcholine released from vagal endings possess inhibitory effects on norepinephrine release from cardiac sympathetic nerves [44], suppressing the adrenergic signaling cascade in ventricular cardiac myocytes [45]. Acetylcholine can act on muscarinic receptors situated in sympathetic presynaptic nerve endings inhibiting the release of norepinephrine [46] [47] [48]. The interaction between the sympathetic and parasympathetic nervous system may also be partially modulated by second messengers in cardiac cells [9] where cholinergic stimulation may inhibit the increase of intracellular cAMP [49] inhibiting the adrenergic signaling cascade [50]. It has been documented that on the sinoatrial node the parasympathetic modulation predominates over the sympathetic activity [50] [51]. This response is known as “accentuated antagonism” [50]. When a vagal electrical stimulation is applied simultaneously to a sympathetic electrical stimulus, there is a reduction in HR similar to that observed in the presence of vagal stimulation alone [52]. Thus, the results from the current study suggest that the parasympathetic improvement produced by PYR in MI mice was able to suppress the augmented sympathetic drive to the heart.

Left ventricle P-V measurements revealed only mild cardiac dysfunction, indicated by increased ventricular volumes and reduced ejection fraction. It has been reported that left ventricular function decreases proportionally with the MI size in mice [1], [33]. Usually, animals with small MI size are excluded from published studies [53], [54]. Nevertheless, in line with our findings, Ahn and co-workers [1] showed that even MI with relatively small size(<20%) displayed significant reduction in ejection fraction. Nevertheless, the size of myocardial infarction in the present study did not affect important indices of ventricular function such as cardiac output (and cardiac index), left ventricular end-diastolic pressure and dP/dt. It is worth noting that Patten and colleagues [35] showed that only the volume and end-diastolic diameter of the left ventricle correlated significantly with the infarct size in mice. Therefore, the slight alterations of cardiac function might be attributed to the small infarct size (∼12%) observed in the present study, while PYR was not able to affect these slight alterations.

Previous studies have shown that electrical stimulation of the vagus nerve improved cardiac function in rats [14] and dogs with heart failure [55]. Moreover, rodent studies using acetylcholinesterase blockers, e.g., PYR and donepezil, described an attenuation of the progression of both cardiac remodeling and cardiac dysfunction, as well as improvement of the long-term survival of animals with heart failure [14], [20], [56], [57]. In addition, Lara and colleagues [11] observed, in mice with reduced expression of the vesicular acetylcholine transporter (VAChT KD), that increased cholinergic activity by means of PYR improved the ventricular dysfunction, indicating the protective role of parasympathetic function in the heart. Nevertheless, in the present study the increased vagal tonic influence to the heart induced by PYR, did not improve the slight cardiac dysfunction exhibited by myocardial infarcted mice.

Therefore, based in the findings of this study, it can be concluded that PYR administration initiated immediately after MI is an important pharmacological approach to protect the alterations in HR and cardiac autonomic imbalance induced by MI.

## Supporting Information

Figure S1 Photomicrographs and bar graphs of the minor diameter of myocytes (yellow lines crossing perpendicular to the nuclei, in hematoxylin and eosin stain) and collagen density (in picrosirius red stain) from the left ventricle of control and myocardial infarcted mice (MI) with or without pyridostigmine (PYR) treatment. Values are presented as the mean ± SEM. Bar = 60 µm.
(TIF)
