# Supplementary figures and images for: Pyridostigmine Restores Cardiac Autonomic Balance after Small Myocardial Infarction in Mice
Source: PLoS One. 2014 Aug 18;9(8):e104476. doi: 10.1371/journal.pone.0104476 (PMC4136726; doi:10.1371/journal.pone.0104476)

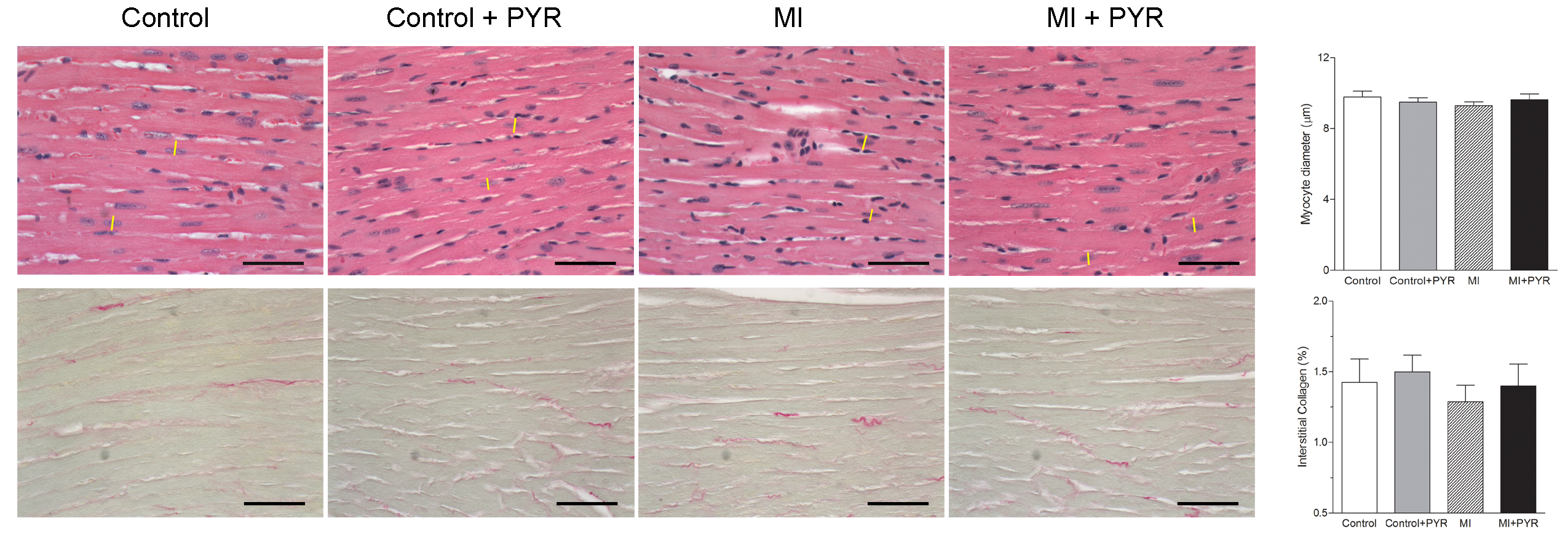

Supplement: Figure S1 — Photomicrographs and bar graphs of the minor diameter of myocytes (yellow lines crossing perpendicular to the nuclei, in hematoxylin and eosin stain) and collagen density (in picrosirius red stain) from the left ventricle of control and myocardial infarcted mice (MI) with or without pyridostigmine (PYR) treatment. Values are presented as the mean ± SEM. Bar = 60 µm. (TIF) [file pone.0104476.s001.tif]
